# Multiphysics analysis of the dual role of magnetoelectric nanoparticles in a microvascular environment: from magnetic targeting to electrical activation

**DOI:** 10.3389/fbioe.2024.1467328

**Published:** 2025-01-07

**Authors:** Martina Lenzuni, Paolo Giannoni, Emma Chiaramello, Serena Fiocchi, Giulia Suarato, Paolo Ravazzani, Alessandra Marrella

**Affiliations:** ^1^ Institute of Electronics, Computer and Telecommunication Engineering (IEIIT), National Research Council (CNR), Milan, Italy; ^2^ Department of Experimental Medicine, Biology Section, University of Genova, Genoa, Italy

**Keywords:** magnetoelectric nanoparticles, multifunctional nanoparticles, extravasation, wireless stimulation, nanotechnology

## Abstract

Minimally invasive medical treatments for peripheral nerve stimulation are critically needed to minimize surgical risks, enhance the precision of therapeutic interventions, and reduce patient recovery time. Magnetoelectric nanoparticles (MENPs), known for their unique ability to respond to both magnetic and electric fields, offer promising potential for precision medicine due to their dual tunable functionality. In this study a multi-physics modeling of the MENPs was performed, assessing their capability to be targeted through external magnetic fields and become electrically activated. In particular, by integrating electromagnetic, fluid dynamics, and biological models, the efficacy of MENPs as wireless nano-tools to trigger electrical stimulation in the peripheral Nervous system present within the dermal microenvironment was assessed. The simulations replicate the blood venous capillary network, accounting for the complex interactions between MENPs, blood flow, and vessel walls. Results demonstrate the precise steering of MENPs (>95%) toward target sites under a low-intensity external magnetic field (78 mT) even with a low susceptibility value (0.45). Furthermore, the extravasation and electrical activation of MENPs within the dermal tissue are analyzed, revealing the generation of high-induced electric fields in the surrounding area when MENPs are subjected to external magnetic fields. Overall, these findings predict that MENPs can be targeted in a tissue site when intravenously administrated, dragged through the microvessels of the venous system, and activated by generating high electric fields for the stimulation of the peripheral nervous system.

## 1 Introduction

Peripheral neuropathies, characterized by abnormalities in the peripheral nervous system (PNS), can emerge due to aging, autoimmune disorders, systemic or metabolic conditions like diabetes, or as a side effect of certain drug treatments, such as chemotherapy (Falvo et al., 2020). These neuropathies can also lead to chronic peripheral pain, often influenced by factors such as poorly fitting prostheses, heterotopic ossification, ulcers, and inadequate wound healing (Cohen et al., 2019; Ferrigno et al., 2020). Even though electrode-implant-based technologies are widely acknowledged to be safe and effective for restoring normal motor function and relieving peripheral pain, they may carry the same risks as any semi-invasive procedure. This includes the hassle of wearing external electrodes, or, even worse, the need to proceed with semi-permanent implants that effectively require surgery (Chiaramello et al., 2022). Furthermore, they are often mechanically incompatible with the soft structures of nerves, leading to potential tissue damage, chronic inflammation, and even scar tissue formation and device failure (Choe et al., 2024). As a consequence, researchers are exploring non-invasive techniques for the establishment of *ad hoc* neurostimulation protocols and treatments of peripheral neurological diseases.

From this perspective, nanoparticles have emerged as non-invasive tools for targeting and treating peripheral neuropathologies. Magnetic field-sensitive nanoparticles, in particular, have been widely studied due to their non-toxicity to the human body (Smith et al., 2023). These nanoparticles can be tracked and visualized using imaging techniques after being conventionally administered via intravenous injection. The ferromagnetic or superparamagnetic properties of these nanoparticles are being exploited in applications ranging from medical imaging to targeted drug delivery and to brain stimulation (Carvalho et al., 2019; Israel et al., 2020).

Among magnetic nanoparticles, “magnetoelectric nanoparticles” (MENPs) show unique properties with respect to all the other nanoparticles known to date: in fact, they can wirelessly induce intrinsic electric fields via remote application of an external magnetic field (Smith et al., 2023). This unconventional feature could provide a non-invasive and targeted method for peripheral neuronal stimulation, with great potentiality in the treatment of neurological disorders. More specifically, MENPs behave like oriented electric dipoles when subjected to external magnetic fields, thus generating local electric fields in the surrounding environment. MENPs can interact with the cellular membrane and activate voltage-gated ion channels, thus triggering neuronal networks (Pardo and Khizroev, 2022; Zhang et al., 2022). The generated electric field intensity is proportional to the applied magnetic field and the dependence of these two is defined through the magnetoelectric coefficient (α) (Smith et al., 2023). Core-shell CoFe_2_O_4_-BaTiO_3_ particles are among the most well-studied magnetoelectric nanostructures in the biomedical field, thanks to their recognized biocompatibility (Smith et al., 2023). Such core-shell configuration maximizes the interfacial coupling between the magnetostrictive core (CoFe_2_O_4_, “CoFe”) and the piezoelectric shell (BaTiO_3_, “BaTi”) (Fiocchi et al., 2022b; Marrella et al., 2023), allowing MENPs to act as wireless interfaces between external devices and human tissues (Sun et al., 2016). MENPs also offer precise control and high spatial resolution, which constitute great advantages over traditional stimulation techniques (Young et al., 2018). As to their deployment to target tissues, recent independent studies have demonstrated the capability to deliver biocompatible MENPs via intravenous injection and their following clearance route (Smith et al., 2023).

To fully exploit MENPs uncovered potentialities, computational models aimed at assessing their behavior in specific tissues come to help. Although these models are essential for translating MENPs into clinical practice, they are currently underutilized (Fiocchi et al., 2022a).

By leveraging on MENPs unique and dual nature, the aim of the present work is to simulate their behavior through numerical models able to (i) target specific regions of the PNS when intravenously injected and (ii) generate electric fields in the chosen region that will potentially stimulate and modulate cell activity. A dermal microcirculation structure, where microvessels of the venous network and nerve terminals coexist, has been considered as target tissue (Cracowski and Roustit, 2020). In this paper, an extensive multiphysics analysis was developed by integrating several components and modules in a multi-level system. Spherical MENPs (100 nm in diameter) have been modeled on the basis of previous research (Marrella et al., 2023). The simulations first evaluated the ability of MENPs with different magnetic susceptibilities to be guided by an external magnetic field within a microvascular network. Then, the following penetration and retention into the surrounding dermal tissue was implemented, by considering the MENPs extravasation out of the blood vessel endothelium into the dermal extracellular matrix. Finally, the portion of MENPs that was able to reach the targeted tissue was subjected to a higher magnetic field to elicit an electric response. The generated field as well as its distribution on the surrounding tissue was quantified, with the aim to predict their stimulation ability. The key findings from these studies will enhance the future implementation of MENPs in biomedical applications, shedding light on their dual role and their potential utilization in peripheral nerve stimulation.

## 2 Materials and methods

COMSOL Multiphysics^®^ 6.1 was adopted for the current study. Different models were developed to precisely simulate the different aspects of the investigation: (i) MENPs magnetic targeting within a capillary fluidic system, (ii) MENPs extravasation, and (iii) MENPs electrical activation within the dermal tissue. Results were analyzed when needed with OriginPro 8.5 (Origin Lab Co., USA).

### 2.1 MENPs magnetic targeting

In the first phase of the study, simulations were conducted to identify the intensity of magnetic fields required to control and target circulating MENPs at a desired location. The modules used in COMSOL Multiphysics^®^ were Magnetic Fields, Laminar Flow, and Particle Tracing for Fluid Flow. The magnetic field intensities were coupled to the laminar flow module. Moreover, the Fluid-Particle Interaction Multiphysics was adopted (coupling Particle Tracing for Fluid Flow and Laminar Flow interfaces). In this way, it was possible to simultaneously model: (i) the distribution of magnetic fields, (ii) the fluid flow, and (iii) the MENPs motion along the fluid flow and under the action of the external magnetic field. Materials properties were found in the published literature, in the Information Technologies in Society (IT’IS) Tissue Properties Database (IT’IS Foundation, 2018), and in the COMSOL built-in library (Table 1).

**TABLE 1:** Properties of materials used in COMSOL Multiphysics^®^ simulations.

Domain	Designation	Value	Unit	References
Blood	Dynamic viscosity	0.0035	Pa*s	IT’IS Foundation (2018), Lenzuni et al. (2023)
	Electrical conductivity	0.7	S/m	Jaspard and Nadi (2002), Philips et al. (2019)
	Density	1,060	kg/m^3^	IT’IS Foundation (2018), Saiko (2022)
	Relative permittivity	76.8	—	IT’IS Foundation (2018)
	Relative permeability	1	—	Hoshiar et al. (2018)
Magnet	Electrical conductivity	1/1.50	µohm*m	COMSOL Library
	Relative permittivity	1	—	COMSOL Library
	Recoil permeability	1.05	—	COMSOL Library
Dermal tissue	Dynamic viscosity	130.61	Pa*s	Holt et al. (2008)
	Electrical conductivity	0.49	S/m	IT’IS Foundation (2018)
	Density	1,109	kg/m^3^	IT’IS Foundation (2018)
	Relative permittivity	72.9	—	IT’IS Foundation (2018)
	Relative permeability	1	—	Marrella et al. (2023)
Air	Electrical conductivity	0	S/m	COMSOL Library
	Relative permittivity	1	—	COMSOL Library
	Relative permeability	1	—	COMSOL Library

#### 2.1.1 Geometrical model

**FIGURE 1:**
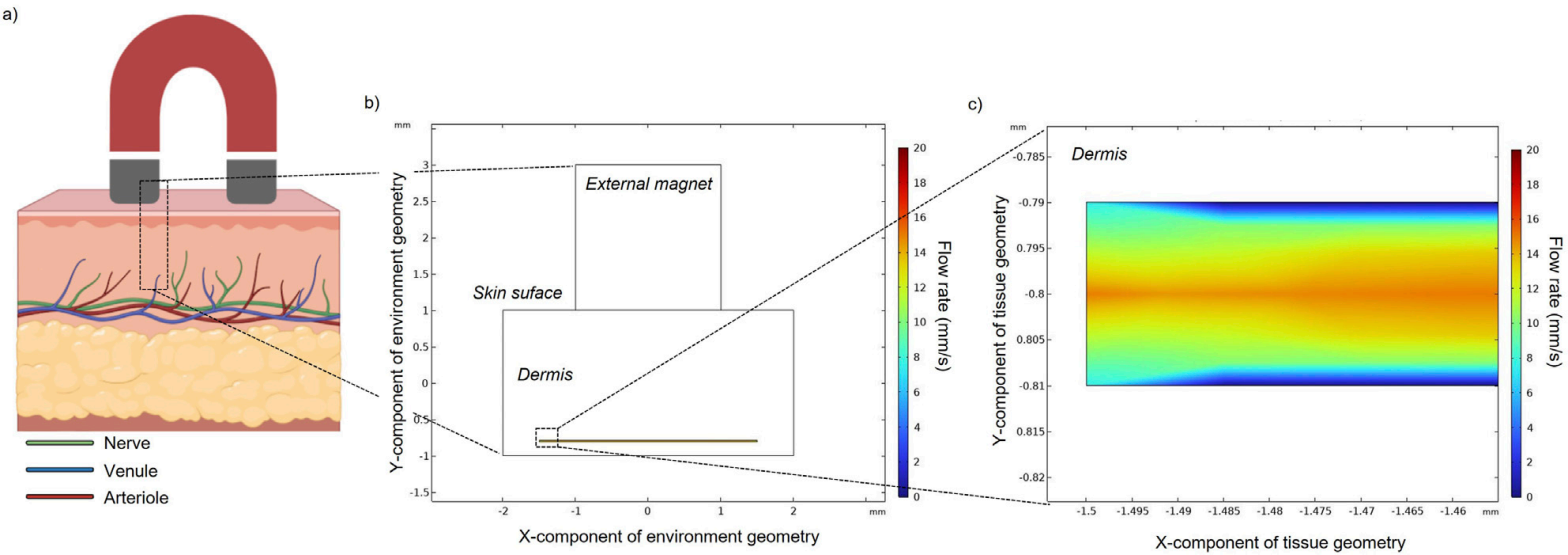
**(A)** Schematic representation of a multilayered skin cross-section highlighting the epidermal (upper layer, dark pink) and dermal (lower layer, light pink) tissues. The dermis contains several types of blood vessels and most of the nerve tissue of the skin. In the proposed model, a magnet is placed on top of the skin surface. **(B)** Simplified constructed geometry in COMSOL with a straight vessel (d = 20 µm) and its flow rate field. **(C)** The enlarged area better shows the vessel diameter and the imposed flow rate.

As depicted in Figures 1A, B, a 2D simplified geometrical model, surrounded by air, was designed and composed of three domains: (i) the blood vessel, (ii) the dermal tissue, and (iii) the external permanent magnet. A blood vessel cross-section (20 µm (height) x 3 mm (length)) was designed and was embedded in the lowest layer of a 2-mm-thick tissue matrix (Cracowski and Roustit, 2020; Saiko, 2022). Here, in particular, the blood vessel considered in the current study simulates a venula located in the upper arm.

#### 2.1.2 Laminar fluid flow

In this model, blood flow is considered as a laminar steady flow of a viscous and incompressible fluid. The motion of blood is considered along the x-component, from the left-handed inlet to the right-handed outlet. Thus, velocity is applied for the inlet boundary with a value of 0.015 m/s, a value consistent with the vessel diameter (Huang et al., 2020; Linninger et al., 2013). No slip condition for all walls was assumed. The continuity Equations 1–3 are applied under the incompressible flow assumption to model the system dynamics:

ρ∇⋅u=0 (1)

$$\rho \left(u\cdot \nabla \right)u=\nabla \cdot \left[-\text{pI}+K\right]+F$$ (2)

$$K=\mu \left({\nabla u+\left(\nabla u\right)}^{T}\right)$$ (3)

where ρ is the fluid density, u the velocity of the fluid, p the fluid pressure, µ the fluid dynamic viscosity, I the unit tensor, T the body temperature (310 K), and F is the force applied to the fluid flow.

#### 2.1.3 Magnetic field

The magnetic field is generated by a permanent magnet located outside the skin. A stationary magnetic field produced by a permanent magnet at a specific location is described by the magnetostatic equations for the static magnetic field derived from Ampere’s law Equations 4, 5:

∇x H=J (4)

∇x A=B (5)

Where H is the magnetic field, J is the current density, B is the magnetic flux density, and A is the magnetic vector potential. The total current density is calculated from Equation 6:

J=σE (6)

Where σ is the electrical conductivity, and E represents the electric field. The magnetic flux density in three different domains of the constructed model (i.e., blood vessel, dermis, and air) is calculated from Equation 7:

$$B=\mu_{0}\mu_{r}H$$ (7)

While in the magnet is evaluated from Equation 8:

$$B=\mu_{0}\mu_{\text{rec}}H+B_{r}$$ (8)

Where µ_0_ and µ_r_ are the magnetic permeability of the vacuum and the selected material, µ_rec_ and B_r_ are the recoil permeability and the remanence (i.e., the flux density when no magnetic field is present), respectively. B_r_ is obtained by multiplying the remanent flux density with a normalized direction field specified in the physics, following Equation 9:

$$B_{r}={∥B}_{r}∥\frac{e}{∥e∥}$$ (9)

Different values of remanent magnetic flux were tested in the simulations in order to calculate the particle targeting percentage.

#### 2.1.4 Particle tracing for fluid flow

MENPs are modeled as spherical solid particles with a diameter of 100 nm. For the studies of magnetic targeting and extravasations, MENPs were modeled as particles consisting of a single material (i.e., only CoFe core), as an approximation. The relative permeability (µ_r_) of the particle material is directly linked to its magnetic susceptibility (χ), as described by Equation 10:

$$\chi =\mu_{r}-1$$ (10)

Since a wide range of magnetic susceptibility values is found in the recent literature for CoFe particles, in the present study three different magnetic susceptibility values (0.45, 3, and 200) were adopted in the models (Betal et al., 2016; Fiocchi et al., 2022b; Marrella et al., 2023). The particle release at the inlet wall has been set to start at t = 1 s and end at t = 2.5 s by steps of 0.5 s, by releasing 60 MENPs each step. The outlet boundary condition was set with particle freeze conditions. Here the particle trajectories are computed with the Newtonian formulation, following Equation 11:

$$F_{t}=\frac{d\left(m_{p}v\right)}{\text{dt}}$$ (11)

Where v is the particle velocity, m_p_ is the particle mass and F_t_ is the total force acting on the particle. Only the major forces are considered in the model, i.e., the hydrodynamic drag and magnetophoretic force (Tehrani et al., 2014). The hydrodynamic drag force F_D_ relies on the Stokes equation and is expressed according to Equations 12, 13:

$$F_{D}=\frac{1}{\tau_{p}}m_{p}\;\left(u-v\right)$$ (12)

$$\tau_{p}=\frac{\rho_{p}\;d_{p}^{2}}{18\mu }$$ (13)

Where u is fluid flow velocity, τ_p_ is the particle velocity response time for spherical particles in Stokes flow, ρ_p_ is the density of the particle, and d_p_ is the particle diameter. The magnetophoretic force (F_MG_) is defined following the Equations 14, 15:

$$F_{\text{MG}}=2\pi r_{p}^{3}\mu_{0}\mu_{r}K\nabla H^{2}$$ (14)

$$K=\frac{\mu_{r,p}-{\;\mu }_{r}\;}{\mu_{r,p}+2\mu_{r}}$$ (15)

Where r_p_ is the radius of the particle, H is the magnetic field strength, µ_r,p_ is the relative permeability of the particles and µ_r_ is the relative permeability of the fluid. The targeting percentage of MENPs on the upper vessel wall was calculated from the ratio of the total number of particles captured and the total number of released particles.

### 2.2 Extravasation of MENPs

Nanoparticles may traverse intercellular spaces and reach the dermal sites where they can be activated in a subsequent step (paragraph 2.3). Here the modeling was focused on the MENPs crossing of the endothelial layer of the capillary blood vessel (Tee et al., 2019). Materials properties are reported in Table 1 (paragraph 2.1.1).

#### 2.2.1 Geometrical model

Endothelial cells were designed in a rectangular shape with constant height (2 µm) and width (50 µm) (Figure 5B). Intercellular gap space was set at 2 µm. Along the upper wall separating the endothelial cells from the extracellular medium/dermis the wall condition for the particles is “pass through”, while along each cell wall, the condition is set to “stick” (i.e., setting then the particle velocity to zero).

#### 2.2.2 Laminar flow and particle tracing for fluid flow

The same equations as described in paragraph 2.1 were used to compute and simulate the laminar flow (2.1.2) and particle tracing (2.1.4). However, two main differences are introduced in this part of the study: (i) number and kinetics of particle release: 10 particles are released from the lower layer of the endothelium (i.e., the upper layer of the blood vessel), every 0.1 s for 3 s (in order to simulate approximately the same number of particles that were previously targeted to the upper vessel wall); (ii) the dermis domain is modeled as a highly viscous matrix where the interstitial fluid (ISF) is responsible for the dragging force (Figure 5A). The drag force experienced by the particles is modeled along the *y*-axis with a velocity of 9*10^−6^ m/s (Ng et al., 2004; Yao et al., 2012). While the density of the domain is similar to the value modeled for blood, the dynamic viscosity is drastically changed (Table 1). Particles’ positions at three different time instants (T1 = 1.5 s, T2 = 3.75 s, T3 = 10 s) were recorded.

### 2.3 MENPs electrical activation

The activation of the MENPs once they are extravasated and reach the dermis is modeled in two steps. Firstly, a 2D axisymmetric study modeling a core (CoFe)/shell (BaTi) MENP (60 nm core diameter, 20 nm shell thickness) present within the dermal tissue was performed to assess the electric potential assumed when MENPs are stimulated by an external magnetic field able to elicit magnetic saturation at the core. Three different COMSOL Modules were implemented (Magnetic Fields, Solid Mechanics, Electrostatics), together with the related coupled Multiphysics (i.e., Magnetostriction and Piezoelectric Multiphysics). Details about the mathematical equations governing the model are derived from Fiocchi et al. (2022b) *.* Then, the extravasated MENPs present in the dermis were modeled as individual dipoles, by setting the electric potential obtained in the numerical analysis described just above, through the Electric Current Module, and in the same position found from the “extravasation” results (Supplementary Figure S1). All MENPs were hypothesized to be aligned in the same direction, i.e., the one of the external magnetic field. Supplementary Table S1 resumes the material properties of the CoFe core and the BaTi shell, following results from our previous studies (Fiocchi et al., 2022b; Marrella et al., 2023).

## 3 Results

### 3.1 Magnetic targeting

Figures 1B, C shows the velocity of the fluid flow, with the maximum value (15 mm/s) occurring at the center of the channel and the velocity decreasing to zero while moving towards the wall of the channel.

**FIGURE 2:**
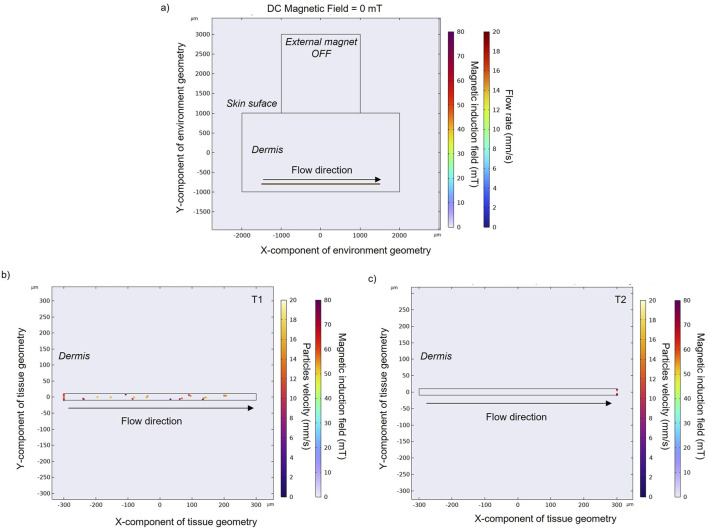
**(A)** Magnetic field obtained without an applied external magnet. Particle positions and velocity without an applied external magnetic field at an intermediate **(B)** and final time point **(C)** in a zoomed section of a small blood vessel located in the human dermis. Black arrows indicate blood flow direction, while spheres represent MENPs.

Flow particles in the absence of an external magnet (Figure 2) only experience the fluidic drag force. The velocity magnitude of MENPs is represented by their colors. Depending on the position of each released particle within the blood vessel, the stream drag force on the particle changes. For example, particles released near the walls of the blood vessel will experience a slower blood velocity due to the flow resistance provided by the no-slip boundary condition. In an intermediate time point (t = 1.5 s) (Figure 2B), the particles are distributed in the channel and some of them have already reached the vessel outlet. At the final time point (Figure 2C), all the particles have been dragged along with the blood flow and are stuck to the outlet boundary of the vessel.

**FIGURE 3:**
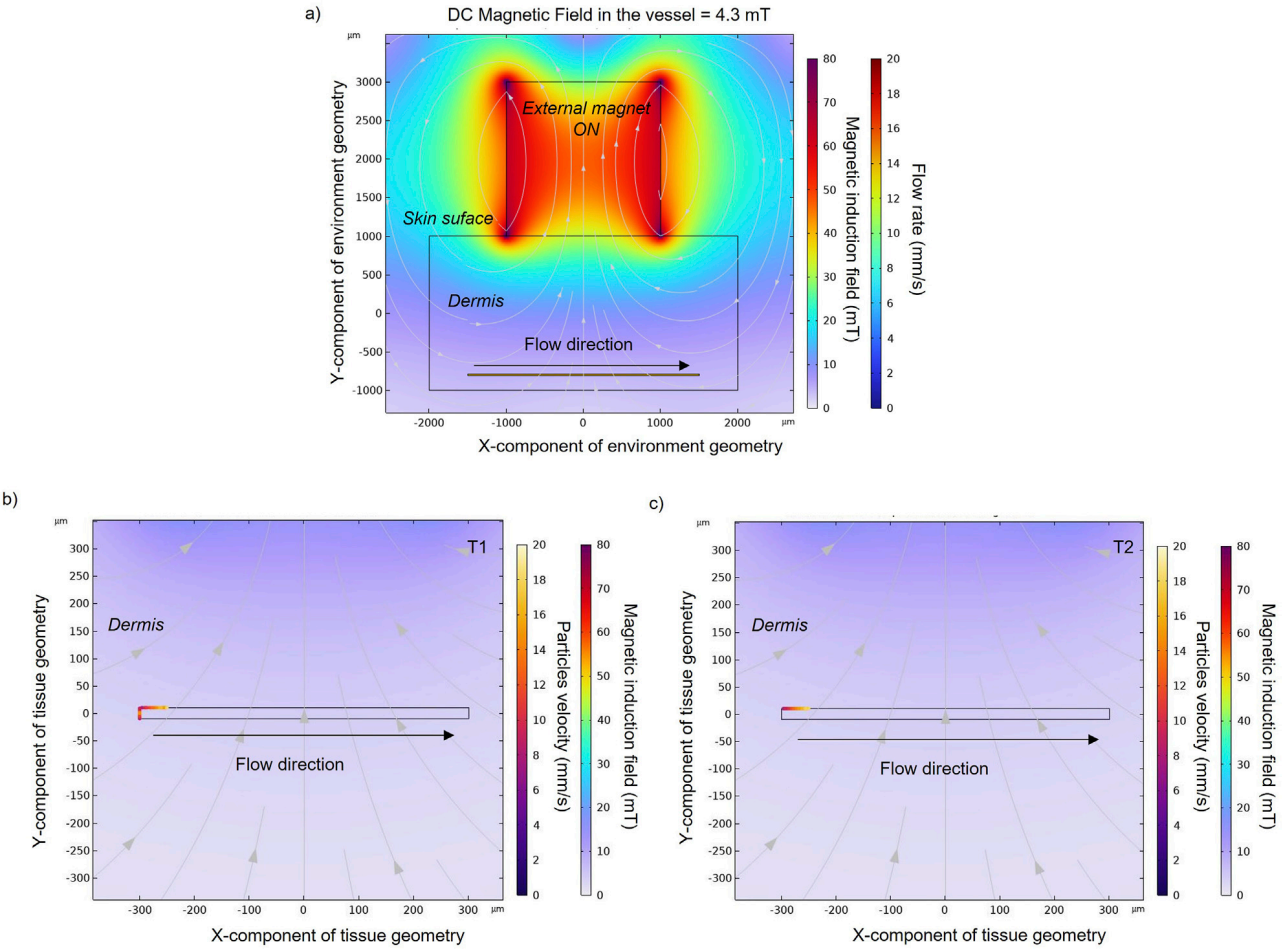
**(A)** Magnetic field obtained with an applied external magnet in order to target the nanoparticles (χ = 0.45) in the upper wall of the blood vessel. Particles positions and velocities with an applied external magnetic field at an intermediate **(B)** and final time point **(C)** in a zoomed section of a small blood vessel. Particle velocity represents the speed of a particle when it hits the vessel wall. Black and grey arrows indicate blood flow direction and magnetic field direction, respectively, while spheres represent MENPs.

Once an external magnet is located over the skin surface, its magnetic force tends to attract the MENPs. Here the results for MENPs with the lower susceptibility value (i.e., 0.45) are reported in Figure 3. The magnetic force causes the magnetic nanoparticles to accelerate as they approach the magnet (with velocity going up to 18.5 mm/s) (Figures 3B, C). Particles reach the upper vessel wall with different velocities, depending on their initial positions: particles that start their navigation on the upper part of the vessel move in layers of slower velocity with respect to those entering the center part of the vessel, and are thereby more easily captured by the magnet.

**FIGURE 4:**
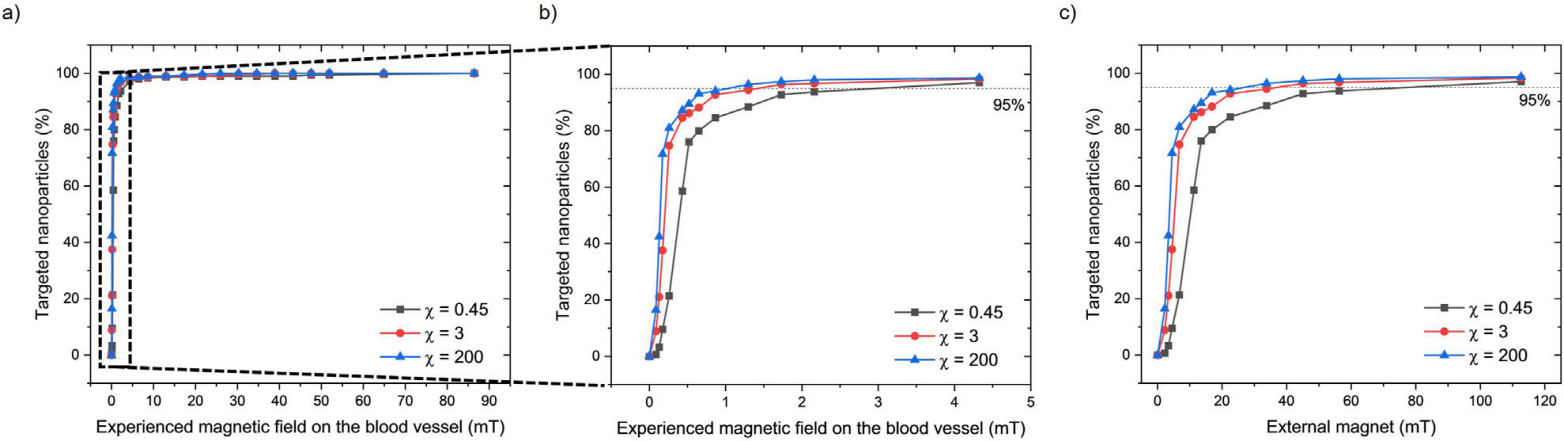
**(A)** The percentage of targeted nanoparticles (with 3 different magnetic susceptibility (χ) values) experiencing different magnetic fields in the upper part of the blood vessel. **(B)** A zoom-in of the dashed area shows the effects of experienced magnetic fields up to 4.5 mT by the nanoparticles. **(C)** The percentage of targeted nanoparticles correlated with the applied external magnetic force from the external magnet.

Due to the high blood flow rate, not all the tested magnetic field values (from 0 to 112 mT) are sufficient to exceed the drag force and capture the particles that are released in the vessel. Data are presented as the percentage of MENPs that reach the upper vessel wall *versus* the magnetic field intensity over the blood vessel segment (Supplementary Table S2). As expected, the targeting percentage of the particles increases with the strength of the magnetic field (Figure 4). A value of 95% targeted particles was achieved with an external magnet of 27 mT, 37 mT, and 78 mT for MENPs with a susceptibility value of 200, 3, and 0.45, respectively (Figure 4C; Supplementary Table S2). On the upper vessel wall (i.e., target location), the average magnetic field strength able to drag the 95% of MENPs was approximately 1.30, 1.73, and 4.33 mT for MENPs with susceptibility values of 200, 3, and 0.45, respectively (Figures 4A, B, Supplementary Table S2). Under the influence of the magnetization force, these particles will remain in the upper vessel wall as long as the magnetic field is applied.

### 3.2 Extravasation

**FIGURE 5:**
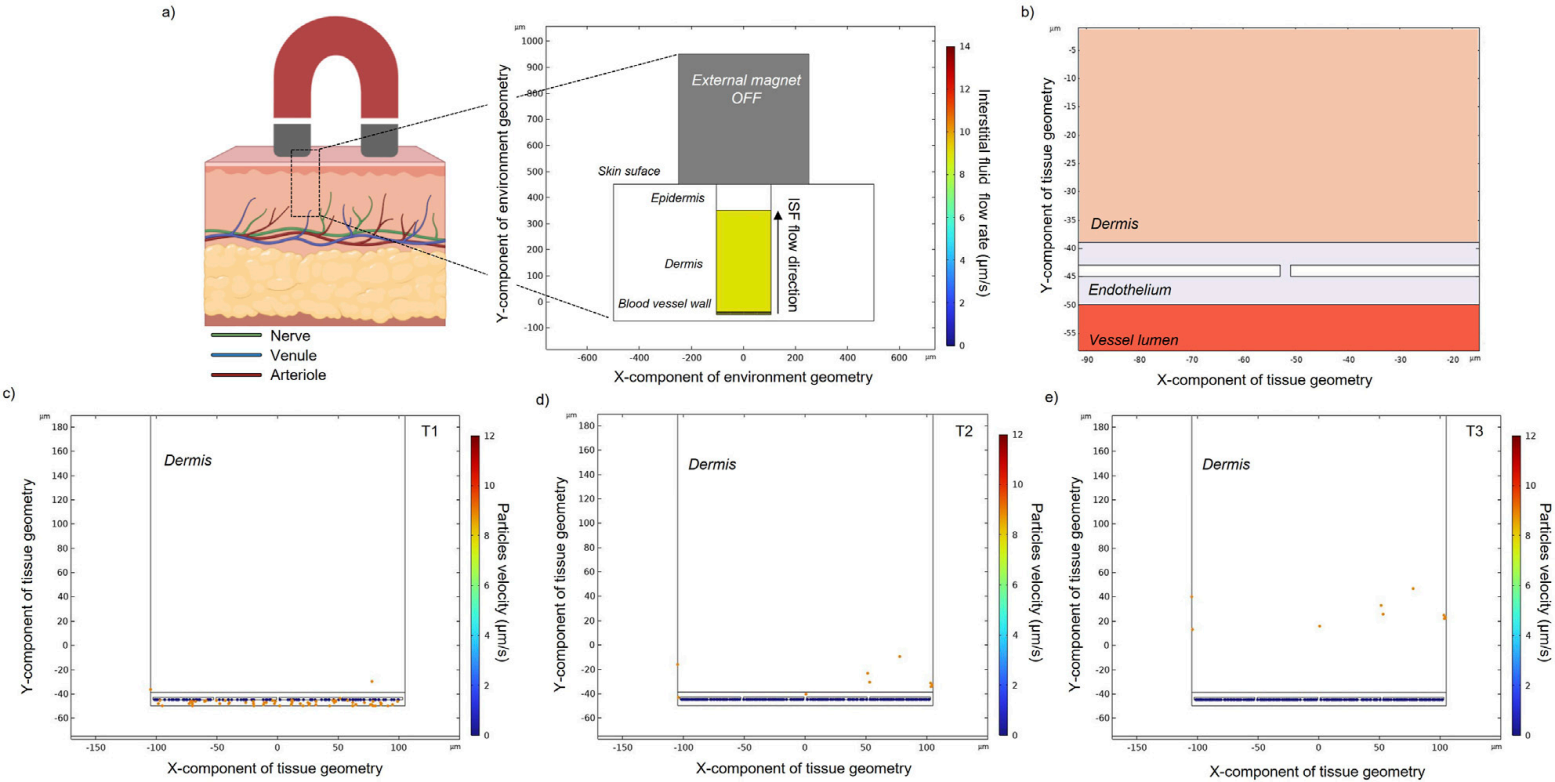
**(A)** Schematic representation of a multilayered skin cross-section, an external magnet and the related COMSOL geometrical model showing the upper blood vessel wall, the dermis, and the epidermis layers. The dermal tissue is permeated by interstitial fluid (ISF) flow, which flows from the deep skin layers to the skin surface. **(B)** Zoom-in schematic of the bio-interfaces between the blood vessel, the endothelium, and the dermal tissue. Particle positions for the depicted regions calculated over time **(C–E)**. Particle velocity represents the speed of a particle when it hits the endothelial cell wall or when it flows into the dermal tissue.

The modeling of particle extravasation was carried out by using the particle tracing modulus in COMSOL to compute particle trajectories in fluids. MENPs (previously targeted to the upper vessel wall) were released from randomized initial locations from the domain vessel lumen (Figure 5C). They were able to travel and enter into the endothelium domain and pass through the intercellular spaces (which were set to five in this model). Their vertical velocity is due to the ISF imposed on the system. Only 4% of the released particles were able to exit through the intercellular spaces, reaching the dermal tissue (Figure 5E).

### 3.3 MENPs activation

**FIGURE 6:**
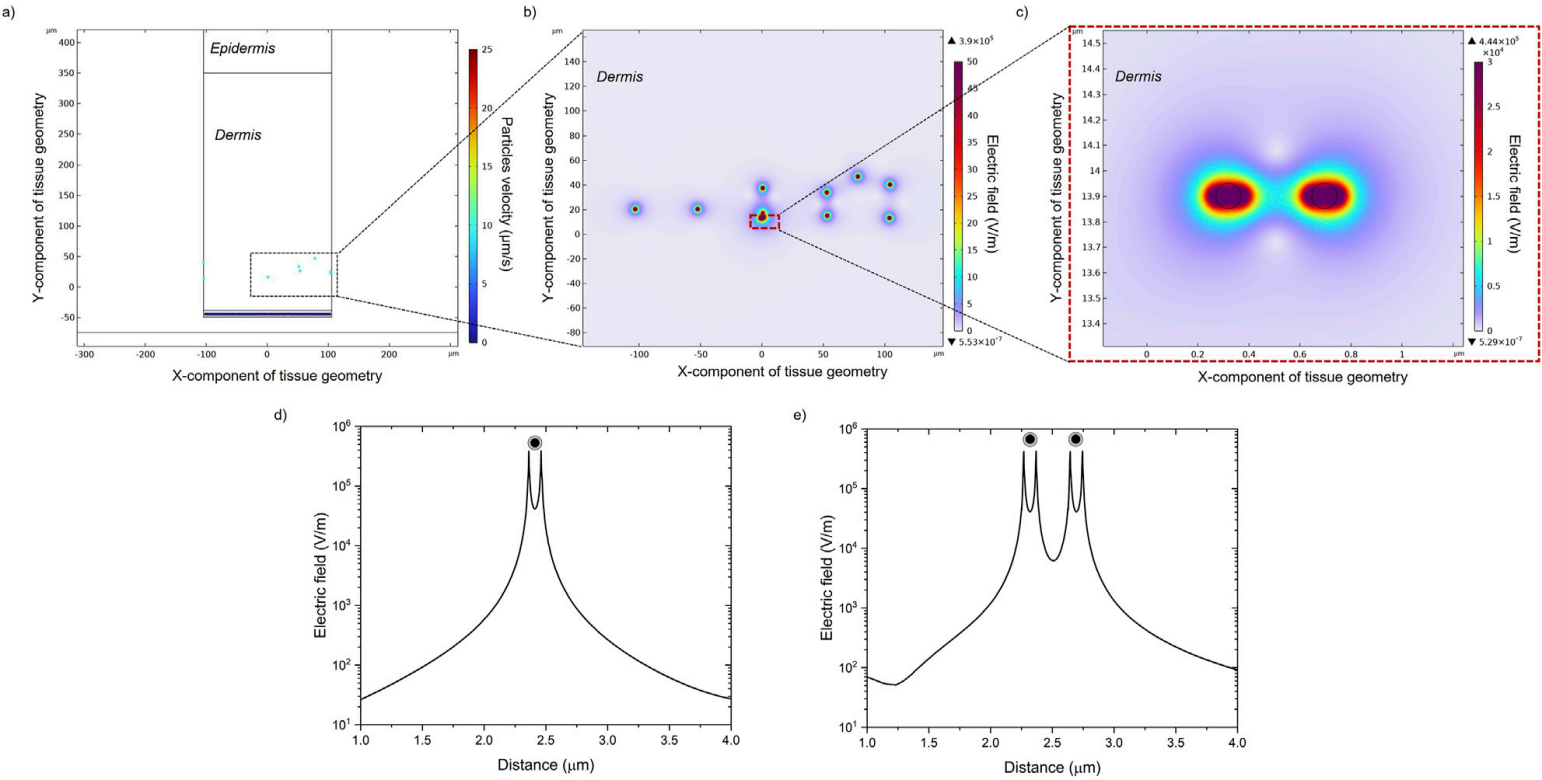
**(A)** Particles positions and velocities in the dermal tissue. **(B)** Electric field distribution generated in the presence of MENPs when a high-amplitude magnetic field is applied. **(C)** A zoom-in image of the dashed box in **(B)** showing the local electric field distribution around two random MENPs. The electric field decays over space around a single MENP **(D)** and two MENPs **(E)**.

At the beginning of the study, MENPs were subjected to a stronger magnetic field (H ≥ Ms) in order to induce their saturation magnetization. The setup is comparable to a magnetic trap that spatially confines particles. The effect of MENPs on electric field distribution within the dermis cross-section was investigated and results are reported in Figure 6. The electric field decay is plotted *versus* the space distance for both a single MENP (Figure 6D) and two MENPs (Figure 6E). Electric fields show a patterned structure depending on how much the particles aggregate and whether they are close to other particles or are alone in the surrounding environment. However, the intensity of the electric field presents an exponential decay with the distance away from the MENP surface. Along an imaginary x plane crossing the core of the selected MENPs (Figures 6D, E), the peak magnitude of the electric field is about 4.4*10^5^ V/m for the two close MENPs and about 3.9*10^5^ V/m for the single MENP (Supplementary Table S3). The coupling effect is responsible for the high electric field intensity in the gap between the two selected MENPs. The highest electric field magnitude is located on the outer shell of the MENPs.

It has to be noted that when the MENPs were exposed to a low magnetic field (H = 4.33 mT, Figure 5) for targeting purposes, they generated a significantly lower electric potential (±2.3*10^−5^ mV) than the one obtained (±1.6 mV, Supplementary Figure S1) when exposed to a saturation magnetic field (H ≥ Ms) necessary for their activation. In a physiological environment, the resulting electric field on the MENPs surface was only a few V/m, which is commonly recognized not to produce an electric field strong enough to induce any harmful effect on human cells (Kanemaki et al., 2024; Lange et al., 2023).

## 4 Discussion

Despite extensive research on magneto-electric nanoparticles and their applications, there is a lack of studies in the current literature focusing on the use of MENPs specifically for peripheral nerve stimulation and regenerative rehabilitation of damaged tissues. The presented multiphysics analysis paves the way for future experimental studies and technological advancements in non-invasive neurostimulation treatments, leveraging the dual role of magnetoelectric nanoparticles. CoFe_2_O_4_-BaTiO_3_ core-shell MENPs can be delivered into the blood circulation via intravenous injections in order to reach their target destination (Hadjikhani et al., 2017; Nguyen et al., 2021). A blood vessel resembling a venula in the upper part of the dermal tissue was modeled, considering that it is the spontaneous path that MENPs follow after intravenous administration before reaching the blood flow microvascular ramification within the dermal tissue where inner peripheral nerves are present (D’Agata et al., 2018). MENPs were initially modeled for magnetic targeting and extravasation studies as single-phase particles composed of CoFe_2_O_4_. This approximation derives from recent findings from our group that indicate that MENPs with maximum core size (at least 80–100 nm) and minimum shell thickness (20 nm) are superior in terms of magnetoelectric coupling coefficient (Fiocchi et al., 2022b). This suggests that the core volume is predominant with respect to the MENP final volume. Moreover, nanoparticles with a diameter of ∼100 nm can have long circulation times in the blood, as small particles (diameter <10 nm) escape by renal clearance and large particles (diameter >200 nm) are quickly eliminated by the reticuloendothelial system of the spleen and liver (Casallas et al., 2023; Lunnoo and Puangmali, 2015). A MENP size of around 100 nm could offer an optimal balance between effective penetration within the PNS as well as a strong magnetoelectric effect, crucial for therapeutic efficacy. This size also minimizes rapid clearance by the body’s defenses, ensuring better targeting and prolonged activity in the peripheral nervous system.

The effect of the magnetic field on the targeting percentage of the particle is shown in Figure 4. More than 95% of MENPs are targeted on the upper vessel wall under a magnetic field of 1.30, 1.73, and 4.33 mT. The maximal exposure of the magnetic field to the blood vessel microenvironments does not exceed 4.5 mT. It is well established that magnetic fields propagate through the human body without any relevant side effect within the limit of 8 T as established by the US Food and Drug Administration (Choi et al., 2021). The strength of the required external magnet for MENPs is far less than those needed for commonly used magnetic nanoparticles (from 0.5 T to 2.5 T) (Shamsi et al., 2018). The results show that particle susceptibility (i.e., magnetic permeability) only has a small effect on particle targeting, even when a low-strength magnetic field is applied. As an example, for an external magnetic field of 50 mT the targeting percentage is 93%, 97%, and 98%, for particle susceptibility values of 0.45, 3, and 200, respectively. Considerable differences are noted only in the left-handed part of the graph (Figure 4C) with an external magnet of <20 mT, corresponding to approximately 1 mT magnetic field within the vessel. It has to be noted that such an applied magnetic field rapidly decays with the distance, ensuring that the magnetic force exerting on the MENPs remains highly localized. This spatial confinement prevents unintended targeting and/or activation of MENPs in distant or unwanted areas. Consequently, there is minimal risk of harmful effects on blood, nerves, muscles, or organs.

When MENPs reach the desired location, they most likely penetrate through the vessel wall and surrounding tissue. Extravasation is a complex phenomenon, still not well understood at a physiological level for nanoparticles. The existence of native large permeable gaps between the endothelial cells of dysfunctional blood vessels (e.g., in the case of inflammation and neurodegenerative diseases) has been widely documented in the literature (Kubíček et al., 2010; Tang et al., 2022). Small particles (diameter <200 nm) can in fact be easily pulled through the pores of the endothelium by an exerted magnetization force (Haverkort et al., 2009; Saxena et al., 2015). Once MENPs are extravasated from the blood vessels to the dermal site, they are transported through the dense interstitial space and extracellular matrix in the lowermost layer of the dermis, which is composed of 70% ISF over its total volume (Samant et al., 2020). It must be underlined that upon removal of the external magnetic field, the nanoparticles will be governed solely by physiological forces, i.e., for MENPs that have successfully extravasated, their movement will be constrained by the properties (e.g., tissue stiffness) of the surrounding tissue, such as dermal extracellular matrix. Consequently, they may remain relatively stable within the targeted tissue or exhibit limited migration due to the motion of the interstitial fluid.

While in the current literature nanoparticle extravasation and their transport from blood to surrounding tissue have been often modeled simply as diffusion, here the extravasation phenomenon was computed with the particle tracing module (Nacev et al., 2011). Here the numerical data show that the percentage of the MENPS passing through the endothelium is about 4%. This result aligns with previous data from the literature, showing that approximately 10% of intravenously administrated nanoparticles transfer into the brain, and 75%–97% (depending on their diameter) of nanoparticles enter pathological tissue using active processes (e.g., endocytosis) through endothelial cells (Guduru et al., 2015; Sindhwani et al., 2020).

In the latter part of the study, the MENPs-induced electric field generated by a higher external magnetic field (=Ms) is shown. As shown in Figure 6, an intensive electric field is always localized near the surface of MENPs and it presents an exponential decay with the distance from the particle surface. For stimulation purposes, the high magnetoelectric coefficient of MENPs can enable strong magnetoelectrically induced electric fields, thus triggering local nervous stimulation via remote application of a magnetic field. It is expected that if MENPs are located near skin cells or red blood cells during exposure to high-strength magnetic fields, the only potential effect observed could be a reversible, highly localized nano-electroporation. This implies that the only expected impact on these cells will be the temporary permeabilization of their membranes, without any permanent damage (Guduru et al., 2013; Kaushik et al., 2017).

These results keep in line with the bi-modal features of MENPs to be dragged and guided by mild external magnetic field intensity gradients and, only subsequently, activated by a stronger magnetic stimulation. In future experimental *in vitro* and *in vivo* settings, a ring-shaped coils-based stimulation system could be designed with the aim of keeping the MENPs confined within a specific tissue area during their electrical activation, minimizing the risk of their redistribution. It has to be noted that the intensity and distribution of the electric field are strictly correlated to the material, geometry, and distribution of the MENPs, and to the dielectric properties of the surrounding tissue (often characterized by considerable heterogeneity) (Miranda et al., 2014). These results suggest and confirm the ability of MENPs to act as a wireless source of electric fields within the dermal tissue, potentially able to stimulate the surrounding peripheral nerves and enhance functional rehabilitation and chronic pain reduction in patients with peripheral neuropathies.

## 5 Conclusion

The use of magnetoelectric nanoparticles for peripheral nerve stimulation and regenerative rehabilitation of damaged tissues represents a groundbreaking approach that holds significant promise as an innovative procedure to address peripheral neural diseases, both in cases of severe disability and when such diseases modify conditions of the patient’s life, causing pain, and discomfort.

By harnessing the unique tunable properties of magnetoelectric nanoparticles, effectively targeting specific regions of the PNS can be achieved with mild external magnetic fields. This computational study demonstrates the feasibility of the application of 100 nm-MENPs to provide non-invasive stimulation to the peripheral nerves at the level of derma, potentially benefiting individuals with poor-fitting prostheses and peripheral nerve damage. In particular, this study allows to control and optimize the MENPs trajectory analysis in a circulation system, their extravasation, and movement due to the ISF flow, and their activation once they reach the dermal tissue. In particular, simulation results showed that MENPs can be accurately (>95%) guided to target sites within the microvascularization of the venous system under the action of an external magnetic field of mild intensity (<5 mT) and then activated by a higher stimulation, with the final aim to wirelessly stimulate nerves of the PNS.

This advanced understanding paves the way for the development of more effective treatments and interventions for a wide range of peripheral neuropathies and chronic neuropathic pain, allowing to modulate nerve activity, reduce pain signals, and eventually restore prosthesis functionality.

## Data Availability

The raw data supporting the conclusions of this article will be made available by the authors, without undue reservation.
