# Supplementary material for: Multiphysics analysis of the dual role of magnetoelectric nanoparticles in a microvascular environment: from magnetic targeting to electrical activation
Source: Front Bioeng Biotechnol. 2025 Jan 7;12:1467328. doi: 10.3389/fbioe.2024.1467328 (PMC11747017; doi:10.3389/fbioe.2024.1467328)
Supplement: Supplementary file 1 [file DataSheet1.docx]

***Supplementary Material***

**Multiphysics analysis of the dual role of magnetoelectric nanoparticles in a microvascular environment: from magnetic targeting to electrical activation**

**Martina Lenzuni^1*^, Paolo Giannoni^2^, Emma Chiaramello^1^, Serena Fiocchi^1^, Giulia Suarato^1^, Paolo Ravazzani^1^, Alessandra Marrella^1^.**

^1^ Institute of Electronics, Computer and Telecommunication Engineering (IEIIT), National Research Council (CNR), Milan, Italy.

^2^ Department of Experimental Medicine, Biology Section, University of Genova, Genova, Italy.

^*^ correspondence: [martina.lenzuni@cnr.it](mailto:martina.lenzuni@cnr.it)

**Table S1**. MENPs core and shell properties.

| **Domain** | **Designation** | **Value** | **Unit** | **Reference(s)** |
| --- | --- | --- | --- | --- |
| **CoFe (core)** | Core diameter | 60 | nm | This study |
|  | Magnetic saturation | 3.69*10^5^ | A/m | (Marrella et al., 2023) |
|  | Saturation magnetostriction | -200 | ppm | (Marrella et al., 2023) |
|  | Density | 5200 | Kg/m^3^ | (Marrella et al., 2023) |
|  | Relative permittivity | 10 | - | (Carvalho et al., 2018) |
|  | Electrical conductivity | 5.2*10^6^ | S/m | (Marrella et al., 2023) |
|  | Poisson ratio | 0.48 | - | (Marrella et al., 2023) |
|  | Young’s modulus | 230*10^9^ | Pa | (Marrella et al., 2023) |
|  | Magnetic susceptibility | 0.45; 3; 200 | - | (Betal et al., 2016; Fiocchi et al., 2022; Marrella et al., 2023) |
| **BaTi (shell)** | Shell thickness | 20 | nm | This study |
|  | Density | 5700 | Kg/m^3^ | (Marrella et al., 2023) |
|  | Relative permeability | 1 | - | (Marrella et al., 2023) |
|  | Electrical conductivity | 178.5 | S/m | (Marrella et al., 2023) |


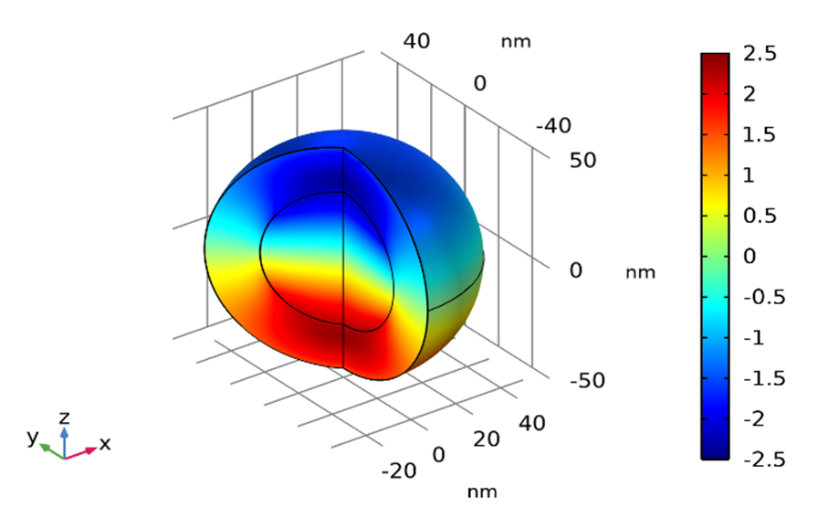


Electric potential (mV)

**Figure S1.** 3D distribution of electric potential (mV), of core-shell magneto-electric nanoparticles, when a high DC magnetic field (H= Ms) is applied in the study.

**Table S2**. Influence of magnetic susceptibility on external magnetic field strength (and the relative magnetic field detected at the blood vessel level) required for different MENPs targeting percentages.

| **Targeted MENPs (%)** | **External magnetic field strength required (mT)** | **Magnetic field detected on the blood vessel (mT)** | **External magnetic field strength required (mT)** | **Magnetic field detected on the blood vessel (mT)** | **External magnetic field strength required (mT)** | **Magnetic field detected on the blood vessel (mT)** |
| --- | --- | --- | --- | --- | --- | --- |
|  | *Magnetic susceptibility (χ): 0.45* | | *Magnetic susceptibility (χ): 3* | | *Magnetic susceptibility (χ): 200* | |
| 25 | 7.21 | 0.28 | 3.65 | 0.14 | 2.65 | 0.1 |
| 50 | 10.23 | 0.39 | 5.29 | 0.20 | 3.66 | 0.14 |
| 75 | 13.41 | 0.52 | 6.95 | 0.27 | 5.34 | 0.21 |
| 95 | 78.11 | 2.97 | 37.06 | 1.43 | 26.95 | 1.04 |

**Table S3**. Electric field strength as a function of distance from the MENPs’ shell. In the two-MENPs configuration, the electric field was analyzed to the right of the rightmost particle.

| **Distance from the MENP shell (µm)** | **Electric field (V/m)** | |
| --- | --- | --- |
|  | *Single-MENP configuration* | *Two-MENPs configuration* |
| 0 | 390000 | 440000 |
| 0.1 | 5055 | 5629 |
| 0.5 | 309 | 461 |
| 1 | 63 | 138 |

**References**

Betal, S., Shrestha, B., Dutta, M., Cotica, L.F., Khachatryan, E., Nash, K., Tang, L., Bhalla, A.S., Guo, R., 2016. Magneto-elasto-electroporation (MEEP): in-vitro visualization and numerical characteristics. Sci. Rep. 6. https://doi.org/10.1038/srep32019

Carvalho, F.E., Lemos, L.V., Migliano, A.C.C., Machado, J.P.B., Pullar, R.C., 2018. Structural and complex electromagnetic properties of cobalt ferrite (CoFe2O4) with an addition of niobium pentoxide. Ceramics International 44, 915–921. https://doi.org/10.1016/j.ceramint.2017.10.023

Fiocchi, S., Chiaramello, E., Marrella, A., Suarato, G., Bonato, M., Parazzini, M., Ravazzani, P., 2022. Modeling of core-shell magneto-electric nanoparticles for biomedical applications: Effect of composition, dimension, and magnetic field features on magnetoelectric response. PLoS ONE 17, e0274676. https://doi.org/10.1371/journal.pone.0274676

Marrella, A., Suarato, G., Fiocchi, S., Chiaramello, E., Bonato, M., Parazzini, M., Ravazzani, P., 2023. Magnetoelectric nanoparticles shape modulates their electrical output. Front. Bioeng. Biotechnol. 11, 1219777. https://doi.org/10.3389/fbioe.2023.1219777
